# Lessons we learned during the past four challenging years in the COVID-19 era: pharmacotherapy, long COVID complications, and vaccine development

**DOI:** 10.1186/s12985-024-02370-6

**Published:** 2024-04-26

**Authors:** Parisa Ghasemiyeh, Soliman Mohammadi-Samani

**Affiliations:** 1 Pharmaceutical Sciences Research Center, Shiraz University of Medical Sciences, Shiraz, Iran; 2 Department of Clinical Pharmacy, School of Pharmacy, Shiraz University of Medical Sciences, Shiraz, Iran; 3 Department of Pharmaceutics, School of Pharmacy, Shiraz University of Medical Sciences, Shiraz, Iran

**Keywords:** COVID-19 variants, Clinical presentation, Pharmacotherapy, Long COVID, COVID-19 vaccines, Vaccine effectiveness (VE)

## Abstract

About four years have passed since the detection of the first cases of COVID-19 in China. During this lethal pandemic, millions of people have lost their lives around the world. Since the first waves of COVID-19 infection, various pharmacotherapeutic agents have been examined in the management of COVID-19. Despite all these efforts in pharmacotherapy, drug repurposing, and design and development of new drugs, multiple organ involvement and various complications occurred during COVID-19. Some of these complications became chronic and long-lasting which led to the “long COVID” syndrome appearance. Therefore, the best way to eradicate this pandemic is prophylaxis through mass vaccination. In this regard, various vaccine platforms including inactivated vaccines, nucleic acid-based vaccines (mRNA and DNA vaccines), adenovirus-vectored vaccines, and protein-based subunit vaccines have been designed and developed to prevent or reduce COVID-19 infection, hospitalization, and mortality rates. In this focused review, at first, the most commonly reported clinical presentations of COVID-19 during these four years have been summarized. In addition, different therapeutic regimens and their latest status in COVID-19 management have been listed. Furthermore, the “long COVID” and related signs, symptoms, and complications have been mentioned. At the end, the effectiveness of available COVID-19 vaccines with different platforms against early SARS-CoV-2 variants and currently circulating variants of interest (VOI) and the necessity of booster vaccine shots have been summarized and discussed in more detail.

## Background

Approximately four years have passed since the first cases of COVID-19 were detected in Wuhan city of China. During these challenging years, various therapeutic options, prophylactic actions, social distancing, and vaccine development have been considered. Despite these efforts especially in the field of patient care and pharmacotherapy, unfortunately, to date (04-05-2024) more than 775 million people have been infected with SARS-CoV-2 and more than 7 million of them died due to COVID-19 infection around the world. Also, according to the epidemiological mortality rate estimation studies, the mortality rate exceeded over 18.2 million till now and many people will lose their lives during this lethal pandemic [1].

SARS-CoV-2 is an enveloped and single-stranded RNA (ssRNA) virus that can bind to its target sites through the angiotensin converting enzyme II (ACE2) receptors. The SARS-CoV-2 recognition, viral fusion, and finally viral entry through the cell membrane can take place via the spike glycoproteins of the virus that call S proteins. These proteins are located on the surface of SARS-CoV-2 that bind to the receptor binding domain (RBD) of ACE2. ACE2 receptors are commonly available in various pulmonary and extra-pulmonary regions including the kidneys, heart, endothelium, and small intestine. Therefore, viral invasion and tissue damage to the lung and all of these organs would be possible during COVID-19 infection. Binding of the SARS-CoV-2 S proteins to ACE2 receptors can lead to cytokine storm and pro-inflammatory processes which in turn can induce tissue damage [2]. Various complications including cardiovascular [3], thrombotic [4], pulmonary [5], and neurological complications [6] have been reported following COVID-19 post infection.

Antiviral drugs that specifically target SARS-CoV-2 can be classified as fusion inhibitors, protease inhibitors, transcription inhibitors, nucleoside reverse transcriptase inhibitors, and those that target the M2 channel protein. Various antiviral agents have been considered for COVID-19 management through the drug repurposing process [7] including remdesivir, favipiravir, ribavirin, umifenovir, lopinavir, ritonavir, molnupiravir, etc [8].. In this regard, remdesivir, an adenosine analog antiviral agent, interferes with RNA-dependent RNA-polymerase (RdRp) which in turn interferes with the viral replication process and has been approved by FDA for COVID-19 management. In addition, molnupiravir, a synthetic nucleoside analog has received FDA approval as an anti-COVID-19 agent in mild to moderate COVID-19 infection [8]. Nirmatrelvir/ritonavir (Paxlovid®) is the first FDA approved oral treatment for COVID-19 which received the emergency use authorization on December 2021 and final approval on approval on May 2023. Paxlovid® tablets (nirmatrelvir 300 mg/ritonavir 100 mg) can be administered orally in non-hospitalized COVID-19 patients with mild to moderate infection who are at high risk of disease progression. Paxlovid® should be initiated within the 5 days of symptom presentation. Non-hospitalized adults and adolescents aged ≥ 12 years and weighting ≥ 40 kg are eligible to receive Paxlovid® during their mild to moderate COVID-19 infection [9].

During the COVID-19 pandemic, we focused on various aspects of COVID-19 pharmacotherapy and different therapeutic options, pharmacokinetic aspects of considered drugs, potential drug-drug interactions (PDDI) among anti-COVID-19 drugs with others, and adverse drug reactions (ADRs) managements in our previous studies [10–15]. In addition, we published our personal experience during the COVID-19 infection and emphasized on the necessity of early initiation of anti-inflammatory agents in patients with mild-to-moderate COVID-19 [16]. In this focused review, at first, the most commonly reported clinical presentations of COVID-19 during these four years have been summarized. In addition, pharmacotherapy aspects and different therapeutic regimens considered in COVID-19 treatment and the latest NIH recommendations on each one have been discussed. Furthermore, the “long COVID” term and its related signs and symptoms have been described. At the end, the effectiveness of available COVID-19 vaccines with different platforms against various SARS-CoV-2 variants and the latest recommendations regarding the necessity of booster dose administration have been summarized and discussed in more detail.

## Clinical presentation

The most commonly reported signs and symptoms during the COVID-19 pandemic with different SARS-CoV-2 variants are summarized in Table 1.

**Table 1:** The most commonly reported complications of COVID-19 infection

Symptoms category	Presentation	Ref.
Respiratory	Cough, rhinorrhea, shortness of breath, lung infiltration, and lung fibrosis	[17–19]
Gastrointestinal	Diarrhea, anorexia, nausea, vomiting, abdominal pain, and digestive problems. GI hemorrhage and bloody diarrhea Bowel ischemia Post-infectious irritable bowel syndrome (IBD) and dyspepsia.	[20, 21]
Hepatobiliary	Acute liver failure, fulminant liver failure with encephalopathy, and scleral icterus. Elevated aspartate aminotransferase (AST) and alanine aminotransferase (ALT). Elevated bilirubin, alkaline phosphatase (ALP), and gamma-glutamyl transferase (GGT). Autoimmune hepatitis (AIH) and primary biliary cirrhosis. Post-COVID-19 cholangiopathy.	[21]
Renal	Electrolyte disturbance especially hyperkalemia, acute kidney injury, renal artery thrombosis, renal infarction, and renal replacement therapy.	[22, 23]
Cardiovascular	Chest pain, ST-elevation acute coronary syndrome, cardiogenic shock, decompensated heart failure, myocardial injury, myocarditis, dysrhythmia, and thromboembolic events.	[24, 25]
Central nervous system	Headache, dizziness, alteration of mental status, ischemic and hemorrhagic cerebrovascular attack (CVA), encephalopathy, meningitis, and seizure.	[26, 27]
Peripheral nervous system	Neuralgia, visual disturbance, dysgeusia, dysosmia, anosmia, ageusia, and Guillain-Barré syndrome (GBS).	[26]
Psychological	Medical trauma, post-traumatic stress disorder, acute stress disorder, cognitive dysfunction, fatigue, depression, social stress and readjustment, anxiety, and psychosis.	[28]
Hematologic	Thrombocytopenia, hemolytic anemia and aplastic anemia.	[29]
Thrombotic complications	Venous thromboembolism (VTE) including deep vein thrombosis (DVT), and pulmonary embolism (PE) Arterial thromboembolic complications including myocardial infarction (MI), stroke, acute limb ischemia, mesenteric ischemia, coronary stent thrombosis. Thrombosis of dialysis catheters in end stage renal disease (ESRD) patients. Extracorporeal membrane oxygenation (ECMO) circuits.	[30]

Results of a comparative study revealed that patients who were infected with the Omicron SARS-CoV-2 variant were younger, had a lower rate of dyspnea, and had a lower rate of hospitalization due to COVID-19 in comparison to those who were infected with the Delta variant. In addition, patients with the Omicron variant infection were more vaccinated and had a lower rate of underlying diseases including obesity. Furthermore, the rate of ICU admission, mechanical ventilation, and in-hospital mortality was significantly higher among patients with Delta variant infection [31].

## Pharmacotherapy

Among all considered therapeutic options, the NIH guideline only recommended remdesivir and ritonavir-boosted nirmatrelvir (Paxlovid®) as preferred agents in non-hospitalized COVID-19 patients. Paxlovid has received FDA approval on 25th May 2023 in non-hospitalized patients with mild to moderate COVID-19, especially those who are prone to develop severe COVID-19 disease. The recommended dose of Paxlovid is 300 mg from nirmatrelvir and 100 mg from ritonavir that should be administered orally twice daily for 5 days [9]. Other therapeutic regimens in this group of patients would be molnupiravir and bebtelovimab. According to NIH COVID-19 Treatment Guidelines, in all non-hospitalized patients, symptom therapy should be considered. In addition, this guideline recommends against the use of dexamethasone or other systemic corticosteroids in non-hospitalized COVID-19 patients. However, in those patients who are at high risk of developing severe COVID-19 infection, Paxlovid and Remdesivir are preferred therapeutic agents, while, bebtelovimab and molnupiravir are considered as alternative therapies when the preferred therapeutic agents are not available [32]. In hospitalized COVID-19 patients who do not require supplemental oxygen therapy dexamethasone or other systemic corticosteroids should not be administered, however in those who are at high risk of progressing to severe COVID-19 infection, remdesivir can be administered. In addition, prophylactic doses of heparin are recommended in these patients [33]. In hospitalized COVID-19 patients who require supplemental oxygen, remdesivir plus dexamethasone can be administered. In addition, in this group of patients therapeutic or prophylactic doses of anticoagulation with heparin should be considered based on the patients’ special condition. In patients who are receiving systemic corticosteroids and still require rapidly enhancing oxygen supply and those with systemic inflammation, baricitinib or tocilizumab can be added to the therapeutic regimen [33]. Finally, in hospitalized COVID-19 patients who require mechanical ventilation or extracorporeal membrane oxygenation (ECMO), dexamethasone plus tocilizumab or dexamethasone plus baricitinib can be administered along with a prophylactic dose of anticoagulation with heparin [34]. In patients who are receiving a therapeutic dose of anticoagulation in the non-ICU setting and then transferred to the ICU, it has been recommended that the therapeutic dose of heparin switch to its prophylactic dose [35]. A summary of the latest recommendations on the most commonly considered therapeutic agents in COVID-19 treatment is summarized in Table 2.

**Table 2:** Latest recommendations on most commonly considered therapeutic agents in COVID-19 treatment

Pharmacologic category	Drug name	Latest recommendations	Ref.
Antiviral drugs	Remdesivir	• Received FDA approval for COVID-19 treatment. • Recommended for the treatment of both hospitalized and non-hospitalized COVID-19 patients. • Remdesivir can be administered without dose adjustment in COVID-19 patients with renal dysfunction (eGFR < 30 mL/min) and also in patients who are on dialysis.	[34, 36–38]
	Ritonavir-boosted nirmatrelvir (Paxlovid)	• Received Emergency Use Authorizations (EUA) from the FDA for the COVID-19 treatment. • Recommended for the treatment of non-hospitalized COVID-19 patients.	[34, 39, 40]
	Molnupiravir	Only should be used in non-hospitalized patients when Remdesivir and Paxlovid are not available.	[34, 39, 41]
Anti-SARS-CoV-2 monoclonal antibodies	Bebtelovimab	Recommended the use for mild to moderate COVID-19 only when remdesivir and Paxlovid are not available.	[34, 42, 43]
	Vilobelimab	Emergency Use Authorization (EUA) by Food and Drug Administration (FDA) to administer within 48 h of mechanical ventilation or extracorporeal membrane oxygenation (ECMO) in hospitalized adult COVID-19 patients.	[44]
	Bamlanivimab plus Etesevimab	Recommended against the use for the treatment of COVID-19 or post-exposure prophylaxis.	[34, 45]
	Casirivimab plus Imdevimab	• Recommended against the use for the treatment of COVID-19 or post-exposure prophylaxis. • Casirivimab plus Imdevimab are not effective against the Omicron variant and should not be used as prophylactic agents.	[34, 46]
	Sotrovimab	Recommended against the use for the treatment of COVID-19 or post-exposure prophylaxis.	[34, 47]
COVID-19 convalescent plasma		Recommended against the use for COVID-19 treatment.	[34, 48, 49]
Cell-Based Therapy	Mesenchymal stem cells	Recommended against the use for COVID-19 treatment except in clinical trials.	[34, 50]
Immunomodulators	Colchicine	Recommended against the use for hospitalized and non-hospitalized COVID-19 patients.	[34, 51, 52]
	Intravenous immunoglobulin (IVIG)	Recommended against the use for the treatment of patients with acute COVID-19 except in clinical trials.	[34, 52]
	Inhaled corticosteroids	Insufficient data available to recommend for or against the use in COVID-19 treatment.	[34, 53, 54]
	Fluvoxamine	Insufficient data available to recommend for or against the use in COVID-19 treatment.	[34, 39, 55]
	Granulocyte-Macrophage Colony-Stimulating Factor Inhibitors	Insufficient data available to recommend for or against the use in COVID-19 treatment.	[34, 56]
	Systemic corticosteroids: Dexamethasone	Recommended for the use in hospitalized COVID-19 patients based on disease severity.	[34, 57–59]
	Inhaled corticosteroids: • Inhaled budesonide plus fluvoxamine • Inhaled fluticasone	Insufficient data available to recommend for or against the use in COVID-19 treatment.	[60]
	IL-1 inhibitors: Anakinra	Insufficient data available to recommend for or against the use in COVID-19 treatment.	[34, 61]
	IL-6 inhibitors: Tocilizumab	Recommended for the use in hospitalized COVID-19 patients based on disease severity.	[34, 62, 63]
	Janus Kinase Inhibitors: Barictinib (or Tofacitinib)	• Recommended for the use in hospitalized COVID-19 patients based on disease severity. • Recommended against the use of baricitinib plus tocilizumab except in clinical trials.	[34, 64, 65]
	Bruton’s Tyrosine Kinase (BTK) Inhibitors (ibrutinib or acalabrutinib)	Recommended against the use for COVID-19 treatment except in clinical trials.	[34, 66]
	Siltuximab	Recommended against the use for COVID-19 treatment except in clinical trials.	[34, 67]
	Canakinumab	Recommended against the use for COVID-19 treatment except in clinical trials.	[34, 68]
Antithrombotic Therapy	Anticoagulant therapy	• Recommended against the use in non-hospitalized COVID-19 patients or continuing in those after hospital discharge. • Unfractionated heparin (UFH) and low molecular weight heparin (LMWH) are superior to oral anticoagulants in hospitalized patients. • Consider the therapeutic doses of heparin in hospitalized non-critically ill COVID-19 patients who receive low-flow oxygen. • Recommended the prophylactic doses of heparin in critically ill COVID-19 patients. • Recommended against the therapeutic dose of rivaroxaban for venous thromboembolism (VTE) prophylaxis or for prevention of COVID-19 disease progression. • Insufficient data available regarding the administration of the therapeutic dose of apixaban for VTE prophylaxis or for prevention of COVID-19 disease progression. • Recommended against the continuation of VTE prophylaxis in COVID-19 patients after hospital discharge	[34, 69–71]
	Antiplatelet therapy	• Recommended against the use in non-hospitalized COVID-19 patients. • Those who are receiving antiplatelet or anticoagulant for an underlying disease should continue therapy during COVID-19 infection.	[34, 72, 73]
Supplements	Vitamin C	Insufficient data available to recommend for or against the use in COVID-19 treatment.	[34, 74, 75]
	Zinc	• Insufficient data available to recommend for or against the use in COVID-19 treatment. • Recommended against the administration of zinc over than recommended dietary allowance (RDA) for COVID-19 prophylaxis.	[34, 75, 76]
	Vitamin D	Insufficient data available to recommend for or against the use in COVID-19 treatment.	[34, 77, 78]

## Long COVID

Long COVID, also known as “post-acute sequelae of COVID-19” (PASC), could be attributed to the persistent sign and symptoms of COVID-19 or organ failure after acute COVID-19 infection. The incidence of long COVID can vary from 10 to 87% in different studies. Both multisystem inflammatory syndrome in children (MIS-C) and multisystem inflammatory syndrome in adults (MIS-A) which are delayed COVID-19 syndromes can also be considered as long COVID [34]. The common symptoms of long COVID and its effect on various organs are summarized in Table 3. Among the listed signs and symptoms in Table 3, headache, fatigue, hair loss, attention deficit, and dyspnea were the most commonly reported ones.

**Table 3:** The common symptoms of long COVID and its effect on various organs

Organ	Long COVID presentations	Ref.
Respiratory tract	Dyspnea, pulmonary fibrosis, extensive alveolar damage, diffuse bilateral alveolar damage (DAD), severe respiratory failure, lung tissue collapse, acute respiratory tract system (ARDS), persistent oxygen requirement, persistent cough, pulmonary embolism, pulmonary thromboembolism, and pulmonary vein and artery damage. Clinical presentations would be dyspnea, chest pain, hemoptysis, and cough.	[79–81]
Cardiovascular system	Myocarditis, myocardial infarction, thromboembolism, arrhythmia (atrial fibrillation, supraventricular tachycardia), heart failure, hypertension, thromboembolism and hypercoagulability state, microangiopathy, capillary damage, and sudden cardiac death.	[81–84]
Hematologic system	Elevated D-dimer, coagulopathy, immunothrombotic state, thrombocytopenia, altered vascular hemostasis, intra-vascular coagulation, venous thromboembolism (DVT and PE), thromboembolic and hemorrhagic events.	[79, 85]
Renal tract	Decreased eGFR, acute kidney injury (AKI), chronic kidney disease, and kidney infarction due to thromboembolism	[79, 86–88]
Gastrointestinal tract	Diarrhea, constipation, nausea, vomiting, anorexia, abdominal pain, gastroesophageal reflux disorder (GERD), gastrointestinal hemorrhage, increased hepatic inflammatory markers (aminotransferase aspartate (AST), alanine aminotransferase (ALT)), bowel damage, bowel necrosis, small bowel dilation, edema, and mesenteric node hemorrhage	[79, 81]
Central nervous system (Neuropsychiatry)	Mood changes, anxiety, depression, post-traumatic stress disorder (PTSD), sleep difficulties, cognitive dysfunction and memory loss, headache, fatigue, muscle weakness, confusion, and hypoxic brain injury.	[81, 87]
Skin	Maculopapular exanthem (morbilliform), papulovesicular rash, red purple papule, urticaria, pernio-like lesions, and hair loss.	[87]
Endocrine system	Hyperglycemia and impaired glucose tolerance, new onset diabetes mellitus, and starvation ketoacidosis.	[81, 89]

## Currently circulating SARS-CoV-2 variants

### Currently circulating variants of concern (VOCs)

Fortunately, there is no currently circulating SARS-CoV-2 variant that fulfill the VOC criteria.

### Currently circulating variants of interest (VOIs)

Some of the Omicron lineages including XBB.1.5, XBB.1.16, and EG.5 are considered as currently circulating VOIs.

#### XBB.1.5

XBB.1.5 lineage was first detected in the United States of America (USA) and have the spike mutations of N460K, S486P, and F490S. The transmissibility, immunity, and disease severity of this lineage is similar to the baseline [90]. Therefore, XBB.1.5 has no additional public health risk in comparison to other currently circulating Omicron variants and showed a low level of risk of growth rate and disease severity with a moderate risk of antibody escape [91]. The neutralizing activity of the available mRNA vaccines against XBB.1.5 lineage has been reduced in comparison to the early Omicron lineages [92].

#### XBB.1.16

XBB.1.16 is an Omicron lineage that has a genetic profile similar to XBB.1.5 lineage. XBB.1.16 has no additional public health risk in comparison to other currently circulating SARS-CoV-2 variants and lineages. The level of risk of growth rate and antibody escape of XBB.1.16 lineage is moderate. Also, the level of risk of disease severity of this lineage is low [93].

#### EG.5

EG.5 is another Omicron lineage which is a currently circulating VOI. It has shown F456L, N460K, S486P, and F490S spike mutations with similar transmissibility to the early Omicron sublineages. EG.5 has a moderate level of risk of growth rate and antibody escape with a low risk of disease severity. Although the prevalence, growth rate, and antibody escape potential of EG.5 lineage have been enhanced in comparison to the other currently circulating Omicron sublinages, however, the COVID-19 disease severity has not increased significantly. To date, EG.5 Omicron lineage is the most prevalent SARS-CoV-2 variant worldwide [94].

#### BA.2.86

BA.2.86 is a linage of BA.2 Omicron which was first detected in Israel and Denmark on 24th July 2023. This lineage is considered as currently circulating VOI with low public health risk at the global level. BA.2.86 has numerous mutations in the spike protein. Although the BA.2.86 lineage is potential to induce a sudden increase in the number of COVID-19 cases, however, the severity of disease would not be higher. According to the recent studies, convalescent plasma of those who have XBB infection, showed sufficient neutralizing activity against BA.2.86, therefore, it seems that XBB.1.5 monovalent COVID-19 vaccines can be effective against the BA.2.86 lineage. To date, no changes in clinical presentation or the severity of COVID-19 have been reported with BA.2.86 infection [95].

#### JN.1

JN.1 is a sublineage of BA.2.86 with high transmissibility which was first detected on August 2023 and is currently considered as VOI. Nowadays, JN.1 is the most prevalent SARS-CoV-2 sublineage in the world, however, it has low public health risk and no changes in disease severity or hospitalization rate have been reported yet with JN.1 sublineage. Although an immune escape has been reported with JN.1 in comparison to other co-circulating SARS-CoV-2 lineages, however, it seems that the monovalent XBB.1.5 booster vaccine would be still effective against JN.1 sublineage [95].

### Currently circulating variants under monitoring (VUMs)

Some of the Omicron lineages including XBB, XBB.1.9.1, and XBB.2.3 are considered as currently circulating VUMs. There are limited data available regarding the transmissibility, growth rate, immune escape of these new Omicron lineages. The main spike mutations that have been occurred in these lineages are K444T, L452R, E180V, T478R, F486P, I332V, D339H, R403K, V445H, G446S, N450D, L452W, N481K, 483del, and E484K. However, the epidemiological and phenotypic impact of these mutations and related lineages are still remaining unclear and further studies and monitoring are required [90].

## De-escalated SARS-CoV-2 variants

### Alpha

Alpha variant, also known as B.1.1.7, was first detected on September 2020 in the United Kingdom. Alpha variant showed 30% increase in viral transmissibility and infectivity and also could induce severe COVID-19 disease. The key mutations of the Alpha variant were N501Y, H69/V70, P681H, and Y144. Due to these mutations, the effectiveness of vaccines and monoclonal antibodies were reduced against this variant in comparison to wild type SARS-CoV-2. Moreover, the ACE2 binding potential was increased by 10-fold [96, 97].

### Beta

Beta variant, also known as B.1.351, was first detected on October 2020 in the South Africa. Beta variant showed 50% increase in viral transmissibility and infectivity and also resulted in severe COVID-19 disease. The key mutations of the Beta variant were N501Y, E484K, K417N, L18F, D80A, D215G, and A701V. Due to these mutations, the effectiveness of vaccines and monoclonal antibodies were reduced against this variant in comparison to wild type SARS-CoV-2. Moreover, the ACE2 binding potential was increased by two-fold [96, 97].

### Gamma

Gamma variant, also known as P.1 lineage, was first detected on November 2020 in the Japan and Brazil. Gamma variant was accompanied by 30–40% increase in viral transmissibility and infectivity and also resulted in severe COVID-19 disease. The key mutations of the Gamma variant were K417T, E484K, AND N501Y. Due to these mutations, the effectiveness of vaccines and monoclonal antibodies were reduced against this variant in comparison to wild type SARS-CoV-2. Moreover, the ACE2 binding potential was increased by five-fold [96, 97].

### Delta

Delta variant, also known as B.1.617, was first detected on December 2020 in the India. Delta variant was accompanied by > 90% increase in viral transmissibility and infectivity and induced severe COVID-19 disease. Furthermore, the infected patients had much higher viral RNA load in comparison to the previous variants. The key mutations of the Delta variant were R158G, L452R, T478K, D614G P681R, and D950N. Due to these mutations, vaccine effectiveness was reduced, however, the effectiveness was 80% remained against severe COVID-19 disease and hospitalization. Administration of monoclonal antibodies including casirivimab, imdevimab and sotrovimab was associated with reduced hospitalization and mortality rate due to the Delta variant infection. Moreover, the ACE2 binding potential was increased by two-fold [96, 97].

## COVID-19 vaccines effectiveness against early SARS-CoV-2 variants

Due to the several irretrievable complications of COVID-19 and the high mortality rates around the world, the best way to eradicate or to silence this pandemic is prophylaxis through mass vaccination. In this regard, soon after the COVID-19 pandemic occurrence, many scientists around the world focused on vaccine design and development to overcome this pandemic and its related morbidities and mortalities. Therefore, various vaccine platforms including inactivated vaccines, nucleic acid-based vaccines (mRNA and DNA vaccines), adenovirus-vectored vaccines, and protein-based subunit vaccines have been developed [98]. Available COVID-19 vaccines, their effectiveness, and coverage against early SARS-CoV-2 variants are summarized in Table 4. Through the worldwide mass vaccination, the COVID-19 pandemic has been relatively controlled and both the mortality and infection rates have been reduced significantly worldwide which emphasizes on vaccine effectiveness in COVID-19 prevention or reduce its severity. Only in moderately or severely immunocompromised patients who cannot be fully vaccinated, tixagevimab/cilgavimab (300 mg/300 mg) (Evusheld®) can be administered as an IM injection in two consecutive doses for the purpose of SARS-CoV-2 pre-expose prophylaxis [34]. A summary of available COVID-19 vaccines, different vaccine platforms, their safety and effectiveness have been discussed in detail in our previous review [98]. According to the early circulating SARS-CoV-2 variants of concerns (VOCs) especially the Omicron variant, administration of booster doses of vaccines would be essential to protect against severe infection and enhance vaccine effectiveness against both hospitalization and mortality due to COVID-19. Results of recent studies revealed that the efficacy of different COVID-19 vaccines has been diminished against the early Omicron VOCs. The results of recent studies emphasized the necessity of booster dose administration to enhance vaccine protection against the newly emerging Omicron variant and its sublineages. In this regard, results of a recent study performed in England revealed that among individuals who were vaccinated with either Pfizer-BioNTech, Moderna, or Oxford-AstraZeneca vaccines as their first and second vaccine shots and those who received either Pfizer-BioNTech or Moderna vaccine as their booster shots, the vaccine effectiveness was significantly increased after booster dose administration against the BA.1 and BA.2 Omicron sublineages. Furthermore, the results of this study revealed the highest effectiveness of a full-dose Pfizer-BioNTech or a half-dose Moderna vaccine as booster dose options to protect against the Omicron variant infection and its related morbidities and mortalities [99]. In addition, it seems that mRNA-based vaccines can be adjusted more feasibly by inducing some changes in their encapsulated S-protein to enhance their effectiveness against the early circulating BA.4 and BA.5 Omicron VOCs to avoid the occurrence of further COVID-19 waves with these sublineages predominance around the world. The BA.2.12.1, BA.4, and BA.5 Omicron sublineages showed higher transmissibility over the BA.1 and BA.2 Omicron sublineages. In addition, higher neutralization evasion was reported with BA.2.12.1 BA.4/BA.5 sublineage in comparison to BA.2 after 3-dose COVID-19 vaccination. Furthermore, it seems that the BA.1-derived booster vaccines might not induce broad-spectrum protection against the newly emerging BA.2.12.1 and BA.4/BA.5 Omicron sublineages [100]. It has been reported that the BA.4/BA.5 subvariants were 4.2 folds more resistant against boosted vaccinated individuals in comparison to the BA.2 subvariant [101]. The BA.4/BA.5 neutralizations against the sera of triple-dose vaccinated individuals with either Pfizer-BioNTech or Oxford-AstraZeneca vaccine were reduced significantly in comparison to the previous BA.1 and BA.2 subvariants. The higher viral escape of BA.4/BA.5 can be attributed to the L452R and F486V mutations [102]. Therefore, design and development of some modified COVID-19 vaccines, especially mRNA-based vaccines, with higher vaccine effectiveness and higher protection against these new subvariants would be crucial to avoid further large COVID-19 waves around the world. In this regard, new bivalent mRNA vaccines (Moderna and Pfizer-BioNTech bivalent COVID-19 vaccines), which are containing two mRNA components of ancestral and BA4/BA5 sublinages, have been designed and developed to enhance vaccine effectiveness against the recent SARS-CoV-2 lineages [103].

**Table 4:** Available COVID-19 vaccines, their effectiveness, and coverage against different SARS-CoV-2 variants

Vaccine platform	Company name (Vaccine name)	Vaccine effectiveness and coverage against different SARS-CoV-2 variants	COVID-19 vaccines’ side effects	Ref.
mRNA-based vaccines	Pfizer-BioNTech (BNT162b2)	• Vaccine effectiveness of 95% against wild-type SARS-CoV-2 after two doses of vaccine administration • Vaccine effectiveness of 89% against Alpha variant • Vaccine effectiveness of 87% against Beta variant • Vaccine effectiveness of 88% against Gamma variant • 2.9- to 13.4-fold reduction in serum neutralizing antibodies titers against Epsilon variant in comparison to the wild-type virus • 8-fold reduction in serum neutralizing antibodies titers against Kappa variant in comparison to the wild-type virus • 2.5- to 4-fold reduction in serum neutralizing antibodies titers against Lambda variant in comparison to the wild-type virus • 3.1-fold reduction in serum neutralizing antibodies titers against Mu variant in comparison to the wild-type virus • Vaccine effectiveness of 84–88% against Delta variant	Pain, swelling, redness, fatigue, headache, fever, chills, vomiting, diarrhea, muscle pain, joint pain, lymphadenopathy, shoulder injury, right axillary lymphadenopathy, and right leg paresthesia, allergic reactions including anaphylaxis, paroxysmal ventricular arrhythmia, and syncope.	[104–111]
	Moderna (mRNA-1273)	• Vaccine effectiveness of 94.1% against wild-type SARS-CoV-2 after two doses of vaccine administration • Vaccine effectiveness of 92% against Alpha variant • Vaccine effectiveness of 89% against Beta variant • Vaccine effectiveness of 89% against Gamma variant • 2.8-fold reduction in serum neutralizing antibodies titers against Epsilon variant in comparison to the wild-type virus • 8-fold reduction in serum neutralizing antibodies titers against Kappa variant in comparison to the wild-type virus • 2.5- to 4-fold reduction in serum neutralizing antibodies titers against Lambda variant in comparison to the wild-type virus • 3.1-fold reduction in serum neutralizing antibodies titers against Mu variant in comparison to the wild-type virus • Vaccine effectiveness of 95% against Delta variant	Pain, swelling, redness at the site of injection, fever, fatigue, headache, chills, vomiting, arthralgia, myalgia, urticaria. (These clinical symptoms were mild to moderate after the first dose of vaccine and moderate to severe after the second dose of vaccine), Allergic reactions including anaphylaxis, facial swelling, and Bell’s palsy.	[98, 105, 107–109, 112–114]
Adenovirus-vectored vaccines	Oxford/AstraZeneca (ChAdOx1-S)	• Vaccine effectiveness of 79% against wild-type SARS-CoV-2 after two doses of vaccine administration • Vaccine effectiveness of 91% against Alpha variant • Vaccine effectiveness of 64–67% against Delta variant	Chills, fever, headache, nausea, vomiting, diarrhea, myalgia, arthralgia, injection site tenderness, pain, warmness, pruritus, bruising, swelling, and erythema, fatigue, malaise, and venous thromboembolism.	[105, 110, 111, 115]
	Johnson & Johnson; (Janssen, Ad26.COV2.S)	• Vaccine effectiveness of 66.9% against wild-type SARS-CoV-2 after a single-dose vaccine administration • Vaccine effectiveness of 70.2% against Alpha variant • Vaccine effectiveness of 51.9–64.7% against Beta variant • Vaccine effectiveness of 36.5% against Gamma variant • 3.2- to 4.9-fold reduction in serum neutralizing antibodies titers against Lambda variant in comparison to the wild-type virus • Vaccine effectiveness of 60% against Delta variant	Chills, fever, headache, nausea, vomiting, diarrhea, myalgia, arthralgia, injection site tenderness, pain, warmness, pruritus, bruising, swelling, and erythema, fatigue, malaise, and venous thromboembolism.	[108, 113, 116–118]
Protein subunit vaccines	Novavax (Nuvaxovid, NVX-CoV2373)	• Vaccine effectiveness of 89.7% against wild-type SARS-CoV-2 after two doses of vaccine administration • Vaccine effectiveness of 86.3% against Alpha variant • Vaccine effectiveness of 51.1–60% against Beta variant	Headache, fatigue, and malaise.	[118–120]
Inactivated vaccines	CoronaVac (Sinovac)	• Vaccine effectiveness of 51% against wild-type SARS-CoV-2 after two doses of vaccine administration • Vaccine effectiveness of 59–60% against Delta variant	Injection site pain.	[114, 121, 122]
	Sinopharm (BBIBP-CorV)	• Vaccine effectiveness of 79% against wild-type SARS-CoV-2 after two doses of vaccine administration • Vaccine effectiveness of 46.8% against Gamma variant	Injection site pain and fever.	[123]
	Bharat Biotech (COVAXIN, BBV152)	• Vaccine effectiveness of 81% against wild-type SARS-CoV-2 after two doses of vaccine administration • Vaccine effectiveness of 65.2% against Delta variant	Injection site pain, fever, fatigue, nausea, and vomiting.	[105, 114]

## COVID-19 vaccines effectiveness against currently circulating SARS-CoV-2 variants

About one year ago on 31st August 2022, FDA approved the emergency use of bivalent mRNA vaccines with enhanced effectiveness against the Omicron variant and its lineages. These bivalent vaccines are composed of two mRNA components of SARS-CoV-2 virus (ancestral and BA.4/5) and therefore can be more effective against the new emerging Omicron lineages including BA4/BA5 [124].

Results of a recent study revealed that the mRNA vaccine effectiveness against the XBB.1.5 Omicron lineage was considerably lower than the wild type or BA.2 Omicron lineage. Therefore, XBB.1.5 could significantly evade the vaccine-induced humoral immunity and administration of bivalent vaccines can improve humoral immune response against the XBB.1.5. lineage as a currently circulating SARS-CoV-2 VOI [125]. Results of another study revealed that administration of a single dose of bivalent mRNA vaccine as a booster shot in those who have received 2 to 4 doses of monovalent mRNA vaccines can significantly enhance effectiveness against XBB.1.5. Omicron lineage [126]. In addition, recently a monovalent XBB.1.5 BNT162b2 COVID-19 vaccine has been approved by CDC as a single-dose vaccine for all individuals ages 6 months and older [127].

There are no sufficient data available regarding the vaccine effectiveness against XBB.1.16 and EG.5 Omicron lineages that are considered as currently circulating SARS-CoV-2 VOIs [128]. However, since only minor changes have been occurred in XBB.1.16 and EG.5 lineages in comparison to XBB.1.5 lineage, it seems that monovalent XBB.1.5 BNT162b2 COVID-19 vaccine would still be effective against these newly emerging VOIs [129].

## Potential drug-vaccine interactions

Among the discussed COVID-19 vaccines and therapeutic options, potential interaction between prednisone and Moderna vaccine (or other equivalent vaccines) has been reported. In this regard, it has been mentioned that in patients who are receiving prednisone, based on the dose and duration of prednisone therapy, response to Moderna vaccine can be diminished. In addition, it has been suggested that the initiation of prednisone therapy in those who are currently vaccinated with Moderna vaccine can be postponed to the next couple of weeks or more. Based on CDC recommendation, prophylactic therapy with analgesics and antipyretics including acetaminophen, aspirin, and other NSAIDs before COVID-19 vaccination should be avoided. However, if pain or fever occurred after vaccination, analgesics and/or antipyretics can be administered when appropriate. In patients who are receiving regular regimens of these agents including daily aspirin intake due to myocardial infarction or stroke, the drug should be continued regardless of vaccine injection. Prophylactic antihistamine therapy prior to COVID-19 vaccination should be avoided. Since antihistamines can’t prevent the potential occurrence of mRNA vaccine-induced anaphylactic reactions and they also may mask the crucial signs of allergic reactions related to the vaccine injection. However, for those who are receiving typical antihistamines for other indications, holding the medication prior to COVID-19 vaccination would not be necessary. Since the approved COVID-19 vaccines are not live vaccines, therefore, there is no potential interaction between antiviral drugs and /or antibiotics with COVID-19 vaccines and vaccine effectiveness cannot be affected by these agents. However, in patients with sign and symptoms of viral and/or bacterial infection, COVID-19 vaccination should be delayed until the patient recovery [130].

## Conclusions

Almost four years have passed since the first cases of COVID-19 in Wuhan city of China and to date (04-05-2024) more than 775 million people have been infected with SARS-CoV-2 and more than 7 million of them died due to COVID-19 infection around the world. During these challenging years, various therapeutic options and prophylactic approaches including COVID-19 vaccination were considered to reduce COVID-19 related mortality and morbidities. COVID-19 infection was associated with various organ involvements including respiratory, cardiovascular, renal, gastrointestinal, hematological, neuropsychological, and dermatological complications. Unfortunately, in many cases some of these complications can be prolonged and may lead to post-acute COVID-19 syndrome or “long COVID” syndrome. Therefore, till now, numerous therapeutic agents have been tried as prophylactic and/or therapeutic agents in COVID-19 management as summarized in Table 2 in order to enhance patients’ survival. Optimum therapeutic regimens should be considered individually based on the specific patients’ condition, underlying diseases, and COVID-19 severity. Although many efforts have been made in pharmacotherapy and disease management, millions of people have lost their lives due to COVID-19. Therefore, prevention through the recruitment of COVID-19 vaccines would be the best way to get rid of the COVID-19 pandemic and its complications. According to Table 4, the vaccine effectiveness has diminished during the previous emergence of SARS-CoV-2 variants. However, among different COVID-19 vaccine platforms, the mRNA-based vaccines including Pfizer-BioNTech and Modernavaccines maintained the highest effectiveness against these variants. Vaccine effectiveness against the currently circulating VOIs, including XBB.1.5, XBB.1.16, and EG.5 Omicron lineages, has also been reduced significantly. However, new bivalent and monovalent mRNA-based vaccines including COMIRNATY Original/Omicron BA.4/5 COVID-19 vaccine and monovalent XBB.1.5 BNT162b2 COVID-19 vaccine have been designed and showed enhanced effectiveness against these newly emerging COVID-19 lineages.

In conclusion, although four years have been passed from the initiation of COVID-19 pandemic, it has not been eradicated completely. Therefore, a comprehensive and updated knowledge regarding various COVID-19 signs and symptoms, differential diagnosis, selection and administration of suitable pharmacotherapy regimen based on disease severity and various stages would be crucial. In addition, detection and differentiation of “long COVID” and its related complications is essential. Fortunately, nowadays COVID-19 has relatively become under-control that is result of the massive worldwide COVID-19 vaccination. Due to the newly emerging SARS-CoV-2 variants around the world especially the Omicron variant and its EG.5, BA.2.86, and JN.1 lineages, the necessity of boosted immunity and administration of newly developed bivalent or monovalent mRNA-based COVID-19 vaccines as booster shots should be highlighted to avoid further new COIVD-19 waves occurrence.

## Data Availability

No datasets were generated or analysed during the current study.
